# Insights into the catalytic properties of the mitochondrial rhomboid protease PARL

**DOI:** 10.1016/j.jbc.2021.100383

**Published:** 2021-02-06

**Authors:** Laine Lysyk, Raelynn Brassard, Elena Arutyunova, Verena Siebert, Zhenze Jiang, Emmanuella Takyi, Melissa Morrison, Howard S. Young, Marius K. Lemberg, Anthony J. O’Donoghue, M. Joanne Lemieux

**Affiliations:** 1 Department of Biochemistry, Faculty of Medicine and Dentistry, Membrane Protein Disease Research Group, University of Alberta, Edmonton, Alberta, Canada; 2 Center for Molecular Biology of Heidelberg University (ZMBH), DKFZ-ZMBH Alliance, Heidelberg, Germany; 3 Skaggs School of Pharmacy and Pharmaceutical Sciences, University of California San Diego, La Jolla, California, USA

**Keywords:** rhomboid protease, membrane protease, mitochondria, intramembrane proteolysis, GlpG, PGAM5, PINK1, Smac/Diablo, CL, cardiolipin, DDM, dodecylmaltoside, GFP, green fluorescent protein, IMM, inner mitochondrial membrane, KO, knockout, PARL, presenilin-associated rhomboid-like protease, MBP, maltose binding protein, MTS, matrix targeting sequence, TM, transmembrane

## Abstract

The rhomboid protease PARL is a critical regulator of mitochondrial homeostasis through its cleavage of substrates such as PINK1, PGAM5, and Smac/Diablo, which have crucial roles in mitochondrial quality control and apoptosis. However, the catalytic properties of PARL, including the effect of lipids on the protease, have never been characterized *in vitro*. To address this, we isolated human PARL expressed in yeast and used FRET-based kinetic assays to measure proteolytic activity *in vitro*. We show that PARL activity in detergent is enhanced by cardiolipin, a lipid enriched in the mitochondrial inner membrane. Significantly higher turnover rates were observed for PARL reconstituted in proteoliposomes, with Smac/Diablo being cleaved most rapidly at a rate of 1 min^−1^. In contrast, PGAM5 is cleaved with the highest efficiency (k_cat_/K_M_) compared with PINK1 and Smac/Diablo. In proteoliposomes, a truncated β-cleavage form of PARL, a physiological form known to affect mitochondrial fragmentation, is more active than the full-length enzyme for hydrolysis of PINK1, PGAM5, and Smac/Diablo. Multiplex profiling of 228 peptides reveals that PARL prefers substrates with a bulky side chain such as Phe in P1, which is distinct from the preference for small side chain residues typically found with bacterial rhomboid proteases. This study using recombinant PARL provides fundamental insights into its catalytic activity and substrate preferences that enhance our understanding of its role in mitochondrial function and has implications for specific inhibitor design.

Mitochondria play an essential role in cellular respiration but also play an equally important role in modulating cell death (1). These functions rely on the selective quality control of mitochondrial protein homeostasis (2) that includes the controlled turnover of regulators in the mitochondria by the mitochondrial intramembrane protease PARL. This enzyme was originally named Presenilin-Associated Rhomboid-Like protease after discovery in a yeast-two hybrid screen (3, 4). PARL cleaves various safeguards of mitochondrial health, including the kinase PINK1 (phosphatase and tensin (PTEN)-induced putative kinase 1) (5, 6, 7) and the phosphatase, PGAM5 (phosphoglycerate mutase family member 5) (8), both of which are known to play roles in mitophagy, the selective removal of damaged mitochondria (9). Hence, PARL has been renamed, and the acronym now corresponds to PINK1/PGAM5 Associated Rhomboid-Like protease (10). Additional substrates of PARL have been identified in a recent proteomic analysis including the proapoptotic factor Smac/Diablo (11). The PARL knockout (KO) mouse exhibits a severe respiratory defect, similar to Leigh’s syndrome, which is a consequence of misprocessing of the nuclear encoded substrate TTC19, a subunit of complex III (12). These PARL KO mice present with a severe motor defect, the loss of gray matter (cell bodies of neurons) in the cortex, and early lethality. The mitochondria of the PARL KO mice have a distinct morphology lacking cristae that precedes neurodegeneration in gray matter (13, 14). When the PARL orthologue Pcp1/Rbd1 is knocked out in yeast, similar disturbances are also observed where the cristae and protein–mtDNA assemblies in the matrix dissipate (15, 16). These studies emphasize the essential nature of the PARL-type proteases for cell viability across evolution.

The PARL protease is a member of the rhomboid intramembrane protease family (17, 18), which are membrane-embedded serine peptidases. Their functions range from cleavage and release of membrane-tethered signaling molecules to membrane protein degradation (17, 19). Regulation of PARL activity at the molecular level is thought to occur *via* posttranslational modifications. Different forms of PARL have been identified in various tissues as a result of processing events; these include a protein with a mitochondrial matrix targeting sequence (MTS), a full-length mature form after removal of the MTS (PARLΔ55), and a truncated form derived from cleavage at residue S77 (PARLΔ77), referred to as β-cleavage (20). Ectopic expression of this truncated form of PARL in tissue culture was shown to alter mitochondrial morphology leading to fusion defects and hence has been suggested to be more active (20). In contrast, it was shown by others that truncation of PARL leads to decreased processing of PINK1 (7). The truncation site at S77 was shown to be phosphorylated in response to stress, an event that influences β-cleavage of PARL and its activity in tissue culture cells (21). The mechanism of this putative regulatory switch remains to be determined, and it has not yet been established if PARLΔ77 is generated by PARL itself or other mitochondrial proteases. Despite this, PARL has not been characterized at the molecular level and thus the importance of β-cleavage in its regulation remains unclear.

Our analysis with human recombinant PARL, comparing full-length and the truncated β-cleavage form, allows us to determine kinetics of substrate cleavage and examine the parameters influencing PARL activity. We evaluated PARL activity using SDS-PAGE and FRET-based fluorescent assays with peptide substrates. We observe that β-cleavage increases the catalytic activity of PARL. When PARL is reconstituted in a lipid environment similar to that found in the inner mitochondrial membrane (IMM), we reveal similar substrate specificities yet at an enhanced catalytic rate of cleavage of all substrate peptides when compared with those measured with the enzyme in detergent micelles. In addition, we observe that PARL activity was increased by cardiolipin (CL). Multiplex substrate profiling reveals a substrate preference for PARL with a bulky side chain Phe in P1, which is distinct from the small side chain residues recognized by most bacterial rhomboids. Together, this work provides characterization of the PARL protease and further extends our mechanistic understanding of this important safeguard of mitochondrial homeostasis.

## Results

### Recombinant human PARL expressed in yeast is active

**Figure 1:**
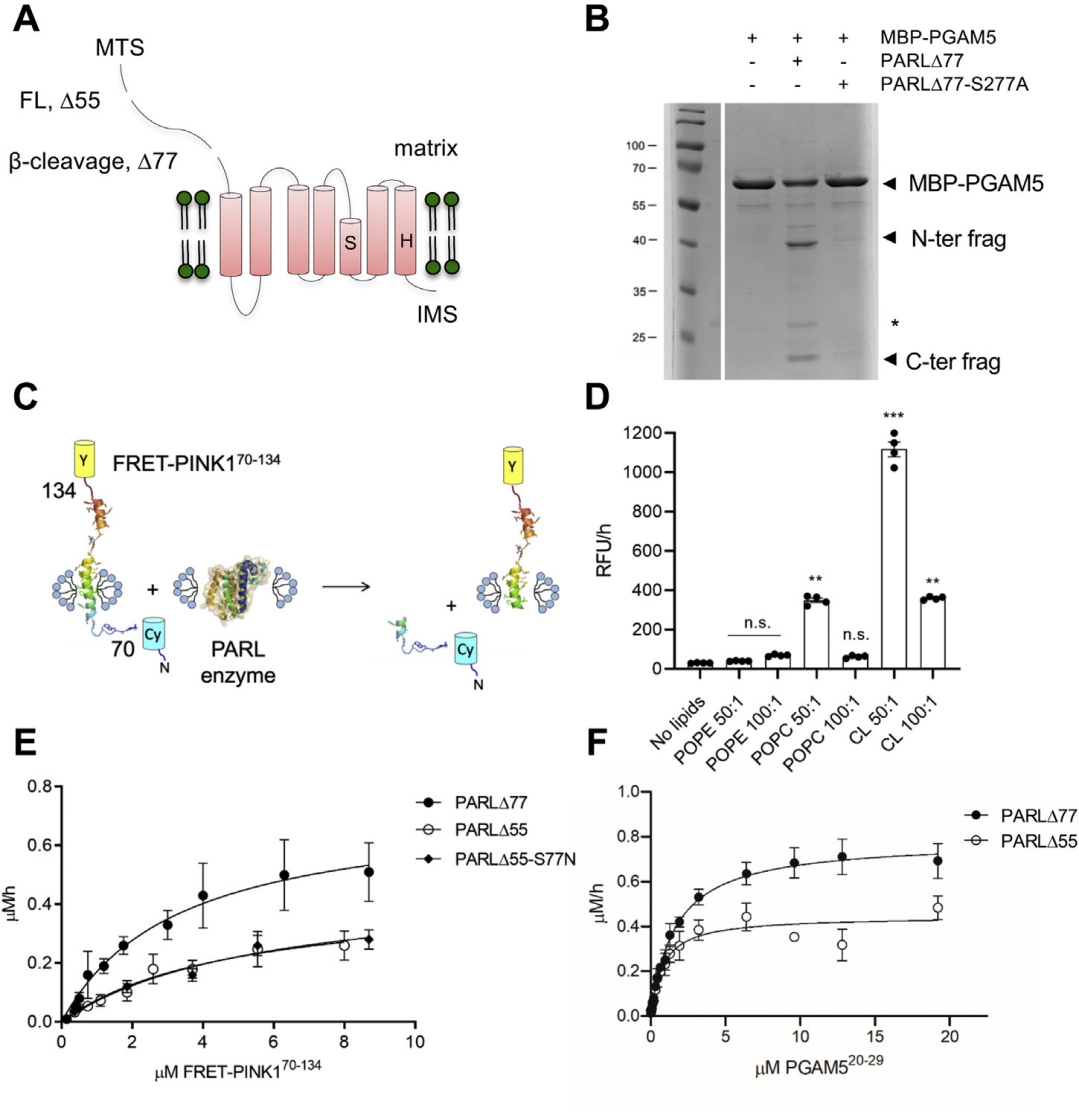
**Recombinant human PARL protease is active.***A*, cartoon representation of the PARL protease topology and truncations. Recombinant human PARL was expressed in *P. pastoris* to generate full-length (FL) starting at residue 55 or the β-cleavage form truncated at residue 77. An inactive PARLΔ77-S277A mutation was also generated. *B*, incubation of recombinant PARLΔ77 with MBP-PGAM5 reveals an expected shift on SDS-PAGE resulting in N- and C-terminal fragments. *Asterisk* in *B* indicates minor contaminant from PARLΔ77 preparation. *C*, a cartoon representation of the substrate construct with residues 70–134 of PINK1 flanked by the CyPet/YPet fluorescence reporter pair. *D*, cleavage of FRET-PINK1(70–134) by detergent-solubilized PARLΔ77 in the presence of increasing lipids. N = 4 ∗ *p* < 0.05; ∗∗ *p* < 0.005, ∗∗∗ *p* < 0.0005. *E*, representative Michaelis–Menten curves for FRET-PINK170–134 cleavage by PARLΔ55, PARLΔ55-S77N, and PARLΔ77 N = 3. *F*, representative Michaelis–Menten kinetic curves for IQ-PGAM5 substrate cleavage by PARLΔ77 and PARLΔ55. N = 3. Values are represented as mean ± SEM. n.s., no significance.

To examine the molecular features that determine PARL activity, we took an approach to express and purify recombinant PARL to study it *in vitro*. Human PARL was cloned into a His-tagged pPICZ expression vector where we added a C-terminal Green Fluorescent Protein (GFP)-tag, which allowed us to monitor protein expression in *Pichia pastoris* (22). The PARL precursor, with the MTS intact, resulted in poor yield (results not shown). The full-length mature form (PARLΔ55), a truncated version representing β-cleavage (PARLΔ77), and a mutant with impaired-β-cleavage PARLΔ55-S77N were successfully expressed (Figs. 1*A* and S1). In addition, the active site mutant PARLΔ77-S277A was also generated. Recombinant PARL proteins were purified using affinity chromatography from dodecylmaltoside (DDM)-solubilized membrane fractions, followed by removal of the GFP-His-tag. Milligram quantities of all PARL variants were obtained using this expression system. The oligomeric states of expressed PARL proteases were examined using SEC, which revealed that PARL existed in a monomeric form in detergent solution (Fig. S2).

To assess if the recombinant PARL was active, we first examined the cleavage of the transmembrane (TM) domain of PGAM5 fused to an N-terminal maltose binding protein (MBP) and a C-terminal Thioredoxin 1 domain (Fig. 1*B*). The approach of using a substrate TM segment fused to MBP was undertaken before for both eukaryotic and prokaryotic rhomboids and the presence of the tag did not affect their ability to cleave the substrates (23, 24, 25). Upon incubation of the MBP-PGAM5 fusion protein with PARLΔ77 in the presence of CL to increase rhomboid activity (see below), new bands, representing the N- and C-terminal cleavage products, were observed on SDS-PAGE. These bands are not present following incubation of MBP-PGAM5 with PARLΔ77-S277A (Figs. 1*B* and S3). This assay confirmed the functionality of human mitochondrial PARL generated in the *P. pastoris* system.

### Lipids enhance PARL activity

To assess the catalytic properties of PARL protease and factors influencing its activity, we designed a FRET-PINK1^70–134^ substrate with fluorophores that were previously used to measure bacterial rhomboid protease activity *in vitro* (26, 27). Residues 70–134 of PINK1, encompassing the predicted TM segment (residues 89–111) and adjacent residues, were cloned between two fluorescent protein reporters, YPet and CyPet, to allow for FRET activity measurement upon cleavage (Fig. 1*C*) (28). Given the fact that PARL protease has never been expressed and studied *in vitro*, all parameters of the activity assay were optimized. First, we examined the pH dependence of PARL activity using FRET-PINK1^70–134^ substrate and determined that the optimum was pH 7.0 (Fig. S4).

Lipids are known to influence membrane protein function (29). In mitochondria, for example, increased CL amounts are observed during mitochondrial stress, which influences protein function at the molecular level (30). Therefore, we assessed the effect of the three primary lipids present in the IMM, namely CL, phosphatidylcholine (POPC), and phosphatidylethanolamine (POPE) on PARLΔ77 activity with the FRET-PINK^70–134^ substrate (Fig. 1*D*) (31). The lipid-free conditions were used as a baseline and consisted of PARLΔ77 reconstituted in detergent (DDM) micelles, while all lipid conditions consisted of a mixed detergent-lipid micelle system. First we assess the effect of different classes of lipids. Compared with conditions with no lipid added, POPE at a molar ratio of 50:1 and 100:1 did not significantly increase PARL activity while POPC at a molar ratio of 50:1 increased activity by fourfold. CL at a molar ratio of 50:1 resulted in a significant increase in PARL activity and to a lesser extent at a molar ratio of 100:1 (Fig. 1*D*). In order to precisely determine the optimal concentration of CL for PARL activity, the initial velocities of PARL-mediated cleavage of FRET-PINK1^70–134^ were measured in the presence of increasing concentrations of CL. The influence of CL on PARL activity resulted in a bell-shaped curve with the 25:1 CL: PARL molar ratio having the most significant effect (Fig. S5). This data indicates that CL modulates the activity of the mitochondrial rhomboid protease PARL by enhancing its overall structural stability through protein–lipid interactions, similar to other mitochondrial proteins (32, 33). Therefore, CL was included in all subsequent protein preparations of detergent-solubilized PARL at the last purification step.

Next, we used this optimized preparation and assay to determine the catalytic parameters of PARL. The cleavage of FRET-PINK1^70–134^ by DDM-solubilized PARLΔ55 and PARLΔ77 in the presence of CL obeyed Michaelis–Menten kinetics (Fig. 1*E* and Table S1) and revealed slow rates of cleavage, 0.43 ± 0.09 h^−1^ and 0.73 ± 0.06 h^−1^, respectively. This, however, reflects the tendency of intramembrane proteases to have slow turnover rates (26, 34, 35).

The truncation of PARLΔ55 to PARLΔ77 was proposed to be autocatalytic (22); however, this has not been confirmed *in vitro*. To ensure the integrity of PARLΔ55 in our kinetic assays, we tested if self-truncation occurred *in vitro* under the conditions of activity measurements. Purified PARLΔ55 was incubated at 37 °C at the concentration of 0.8 mg/ml for 4 h (the longest time used for kinetic assay), and protein samples taken after 2 and 4 h were run on SDS-PAGE (Fig. S6). No additional band corresponding to molecular weight of PARLΔ77 was observed after the time of incubation, suggesting that no autocleavage occurs under these conditions. Furthermore, the catalytic parameters of PARLΔ55S77N, the mutant that prevents β-cleavage, were similar to PARLΔ55, which again suggests that PARLΔ55 does not undergo auto processing since one would expect different catalytic parameters for PARLΔ77 (Fig. 1*E*).

**Figure 2:**
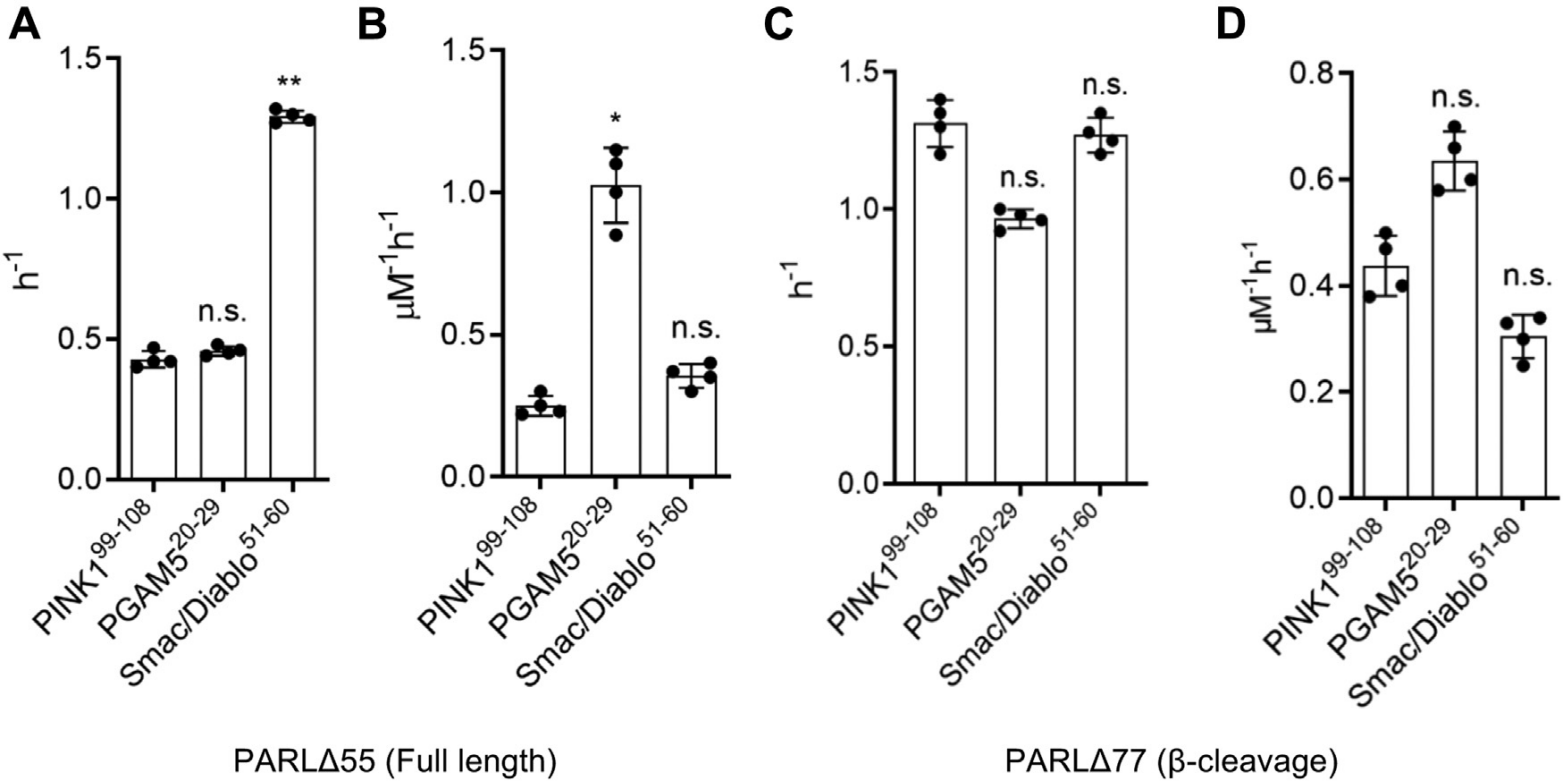
**Catalytic parameters of IQ-peptide substrate cleavage with both PARLΔ55 (full length) and PARLΔ77 (β-cleavage) reveal differences in k**_**cat**_**and k**_**cat**_**/K**_**M**_**values.***A*, turnover rates and (*B*) catalytic efficiency are plotted for PARLΔ55. *C*, turnover rates and (*D*) catalytic efficiency are plotted for PARLΔ77. Experiments were conducted in duplicate with an N = 4. Individual data points (*black dots*) are indicated on the bar graphs, representing the mean ± SEM (∗*p* < 0.05; ∗∗ *p* < 0.005). n.s., no significance.

To further evaluate the cleavage of other known substrates of PARL, we adopted a more facile system to examine cleavage of multiple substrates and generated internally quenched (IQ) peptide substrates (25, 36, 37, 38) based on the amino acids flanking the PARL cleavage sites of PINK1 (39), PGAM5 (8), and Smac/Diablo (11). Kinetic analysis using both full-length and β-truncated PARL with IQ-PINK1^99–108^, IQ-PGAM5^20–29^, and IQ-Smac/Diablo^51–60^ peptide substrates was first performed in detergent, which revealed similar Michaelis–Menten kinetics for all peptides (Fig. 1*F*). The assay allowed us to determine the catalytic parameters for the three primary PARL substrates and examine the substrate specificity (Fig. 2, Tables S2–S4). Similar to previously shown kinetic parameters for FRET-PINK1^70–134^ cleavage, a slow turnover rate for all substrates was observed, with the k_cat_ values ranging from 0.5 to 1.3 h^−1^ with the Smac/Diablo peptide being the fastest cleaved and PGAM5 being the most efficiently cleaved based on k_cat_/K_M_ value. These data also revealed that the turnover rates of PARL proteases, with the short IQ-PINK1^99–108^ peptide and longer FRET-PINK1^70–134^ substrate, were comparable, being, for example, 0.42 ± 0.03 h^−1^ with PINK1 peptide and 0.46 ± 0.09 h^−1^ with FRET- PINK1^70–134^ for full-length PARLΔ55 (Fig. 1*E*, Tables S1 and S2). We conclude that the regions adjacent to the PINK1 cleavage site do not influence the cleavage process, and thus the IQ-peptide substrates are suitable for performing further kinetic studies.

### PARL shows enhanced catalytic rate in liposomes

**Figure 3:**
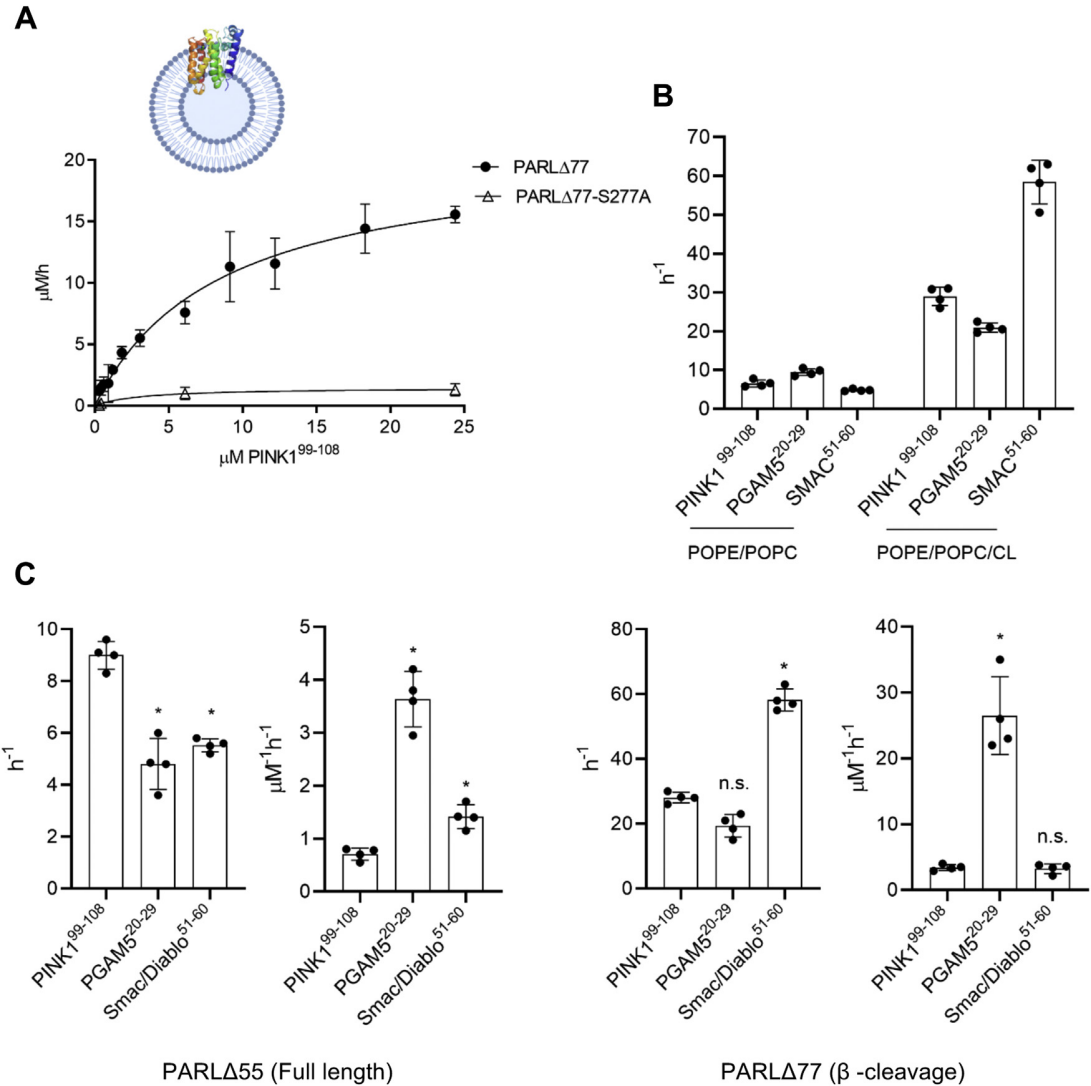
**Enhanced catalytic rate is observed with PARL in proteoliposomes with cardiolipin.***A*, representative Michaelis–Menten curve for PARLΔ77 cleavage of IQ-PINK199–108. *B*, the effect of CL on PARLΔ77 activity in proteoliposomes with IQ-peptide substrates. *C*, bar graphs for turnover rates and catalytic efficiencies of proteoliposome-reconstituted PARLΔ55 and PARLΔ77 with IQ-peptide substrates. Experiments were conducted in duplicate with an N = 4. Individual data points (*black dots*) are indicated on the bar graphs, representing the mean ± SEM (∗ *p* < 0.05; ∗∗ *p* < 0.005). n.s., no significance.

To assess the activity of recombinant PARL toward known substrates in a lipid bilayer, full-length and β-truncated PARL were reconstituted in proteoliposomes (PLs) using *Escherichia coli* lipids that closely resemble the composition of the IMM (31). We determined that the EDANS/Dabcyl - FRET tags prevented incorporation of the substrates into PL; however, the substrate could still be cleaved since the active site is proposed to be near the lipid/water interface (40, 41) analogous to bacterial rhomboid proteases (Fig. 1). To calculate the specific activity of the protease, we quantified the fraction of PARL in the PLs with an outward facing active site using a membrane semipermeable activity-based TAMRA-labeled fluorophosphonate probe (42). In PLs ∼70% of PARL could be labeled with the probe revealing the proportion with the active site in an outward-facing manner (Fig. S7). These values were used to calculate specific activity of the PARL protease. Next, we compared cleavage of three IQ-peptide substrates by reconstituted full-length mature PARLΔ55 and the β-truncated form, PARLΔ77 (Fig. 3, Tables S5 and S6). Overall, we observed that the lipid environment increased the activity of both forms of PARL toward all substrates and the reaction still displayed Michaelis–Menten kinetics (Fig. 3, *A* and *C* and Table S5 and S6); only negligible background activity was detected for inactive PARLΔ77-S277A (Fig. S8). Of all peptides assessed, cleavage of Smac/Diablo by β-truncated PARLΔ77 was the fastest with a turnover rate of 58 ± 7 h^−1^, or 1.0 ± 0.1 min^−1^, and PGAM5 was the preferred substrate with the lowest K_M_ and the highest catalytic efficiency (k_cat_/K_M_) of 26 ± 8 μM^−1^ h^−1^ (Table S6), which agrees with the data obtained in a detergent environment.

To examine if CL influences PARL activity in a membrane environment, we prepared PL samples containing β-truncated PARL and conducted detailed kinetic analysis with the three IQ-peptide substrates (Fig. 3*B*). Two independent PL samples were generated: the first consisted only of POPC and POPE, while the second also contained CL. The turnover rates for the PINK1, PGAM5, and Smac/Diablo peptide substrates with the two PL samples demonstrated a trend similar to that observed in the detergent environment (Fig. 3*C*). When CL was omitted from the PL, we observed two to tenfold slower turnover rates of cleavage for IQ-peptide substrates by β-truncated PARL (Fig. 3*B* and Table S7). This result again demonstrates that CL, which is specific to the inner mitochondrial membrane where PARL resides, influences the proteolytic activity of PARL.

### β-Cleavage influences the activity of PARL

The influence of PARL truncations on activity has been controversial. PARL processing has been proposed to be a regulator of its enzymatic activity (43). Cellular studies with PARLΔ55-S77N, a form of PARL unable to undergo β-cleavage, have shown impaired cleavage of PINK1, with additional data suggesting that the longer form is less active toward PINK1 (7, 21). Another study showed that the β-truncated PARL (PARLΔ77) induces mitochondria fragmentation in cells (20). We used our kinetic analysis to examine whether β-cleavage influences the activity of PARL *in vitro*. Kinetic parameters of full-length and β-truncated PARL in both detergent (Fig. 2, Tables S3 and S4) and PL (Fig. 3, Tables S5 and S6) were determined using three IQ-peptide substrates: PINK1, PGAM5, and Smac/Diablo. In detergent, turnover rates increased (PINK1 and PGAM5) or remained unchanged (Smac/Diablo) with β-truncated PARL when compared with full-length-PARL (Fig. 2). In PL, mimicking a bilayer environment, a similar trend was observed with the turnover rate being significantly enhanced for Smac/Diablo with β-truncated PARL (Fig. 3 and Table S8). The direct influence of PARL truncations on substrate cleavage has not been examined *in vitro* before and with this data we confirm that PARL is catalytically active in either form, thus indicating that processing to the β-cleavage form is not required for proteolytic activity or PARL functionality as was once speculated (7).

### PARL is weakly inhibited by commercial inhibitors

Rhomboids were initially discovered to be serine proteases because the first identified rhomboid protease from *Drosophila*, Rhomboid-1, was sensitive to serine protease inhibitors dichloroisocoumarin (DCI) and tosyl phenylalanyl chloromethylketone (TPCK) (44). Further, the crystal structures of bacterial rhomboids with serine protease inhibitors—diisopropylfluorophosphate and isocoumarins aided in revealing structural insight about their molecular mechanism of catalytic reaction (17). However, the broad-spectrum serine protease inhibitor PMSF does not act on bacterial rhomboid proteases (44, 45, 46). Inhibition of PARL has not been previously explored.

We examined whether inhibitors belonging to three standard serine protease inhibitors families—sulfonyl fluoride (PMSF), chloromethyl ketone (TPCK), and coumarin (DCI)—were able to inhibit PARL when tested with IQ-PINK1 and IQ-PGAM5 substrates. In detergent and PL, for both PINK1 and PGAM5 peptide substrates, we show that PARL activity is not inhibited by 100 μM PMSF, whereas 100 μM TPCK partially inhibits and 100 μM DCI has the largest inhibitory effect (Fig. S9), which is in agreement with previously shown effect for bacterial rhomboid proteases (47). Overall this supports the view that for PARL, specific inhibitors will need to be developed similar to bacterial rhomboid proteases (48, 49).

### PINK1, PGAM5, and Smac/Diablo are competing for the same binding site

With multiple substrates discovered, it is obvious that PARL has pleotropic roles in mitochondria and that substrate cleavage must be precisely regulated (50), whether through compartmentalization (51) or differential substrate binding. It was shown that PARL-mediated differential cleavage of PINK1 and PGAM5 depends on the health status of mitochondria (51). Further studies suggested that the rate of PINK1 cleavage in cells is influenced by PGAM5, indicating that PINK1 and PGAM5 may compete for cleavage by PARL (52). However, it has been speculated that PINK1 and PGAM5 are not competitive substrates *in vivo* since reducing the expression of PINK1 by siRNA did not increase cleavage of PGAM5 by PARL (8), highlighting that function of PARL changes in response to ΔΨ_m_ loss. This raises questions whether PARL substrates bind to the same residues in the active site or alternative binding sites might exist on enzyme’s surface.

**Figure 4:**
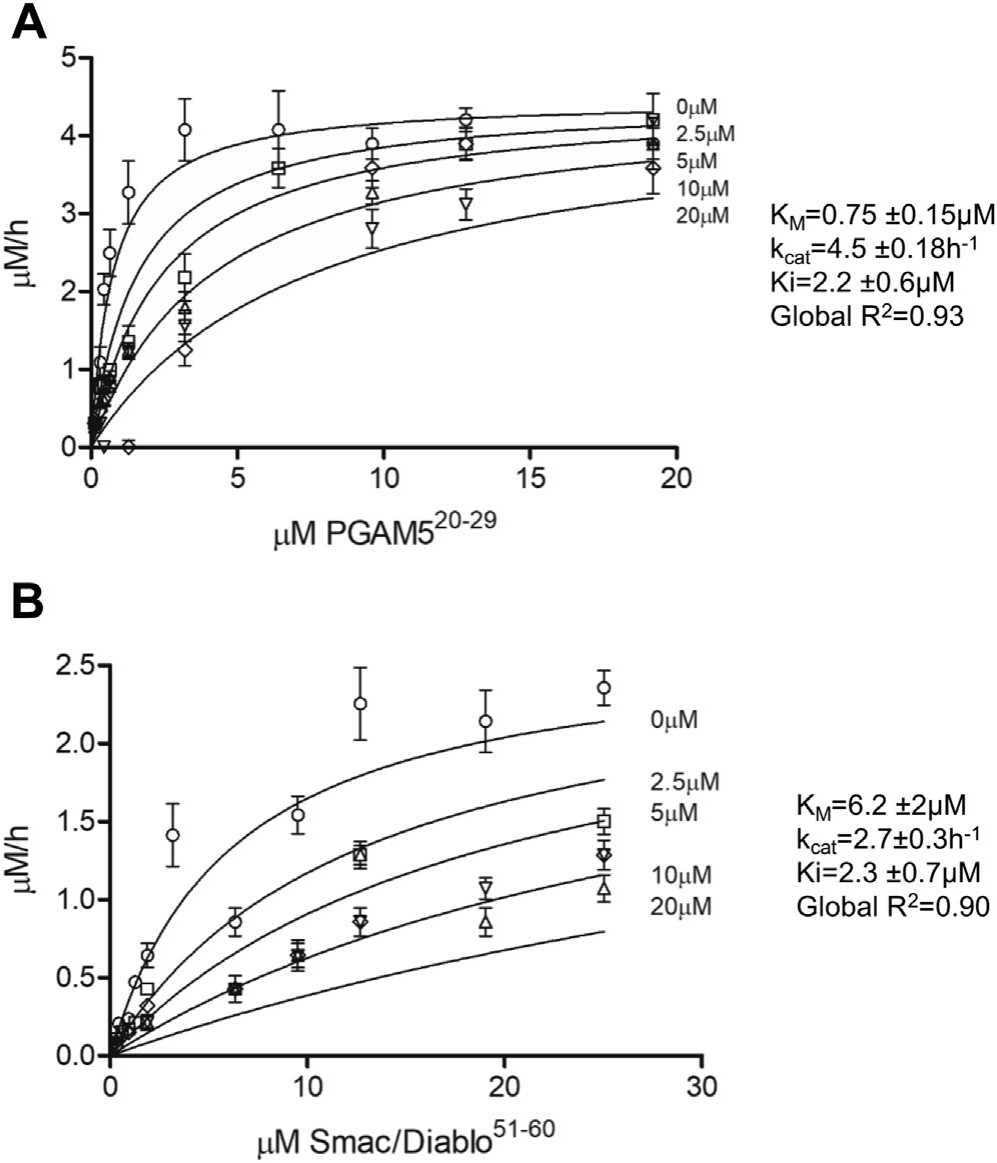
**Competitive studies of PARLΔ77-mediated cleavage of IQ-PGAM520-29 and IQ-SMAC51-60 peptides in the presence of PINK189-111 reveal competitive inhibition.** Cleavage assays of PARLΔ77 (0.8 μM) with: (*A*) IQ -PGAM520-29 (0.13-19 μM) (*B*) or SMAC51-60 (0.3–25 μM), performed in the presence of different concentrations of nonfluorescent PINK189-111 substrate (2.5, 5, 10, 20 μM), reveals competitive inhibition suggestive of identical binding sites. Fluorescence detection of each substrate concentration in the presence of corresponding PINK189-111 without enzyme was used as a negative control. Initial velocities were determined for each substrate concentration. Michaelis–Menten plots were subjected to global fit to distinguish the kinetic model and determine the kinetic parameters. Values are represented as mean ± SEM (N = 3).

To determine if substrates bind to the same site, we performed competition binding assays using fluorescent IQ-PGAM5^20–29^ and IQ-Smac/Diablo^51–60^ as the main substrates and nonfluorescent PINK1^89–111^ (^89^AWGCAGPCGRAVFLAFGLGLGLI^111^) as a competing substrate (Fig. 4). The longer version of substrate was used in this assay in order to reveal possible exosite interactions of substrate with PARL, assuming that regions surrounding the cleavage site might be involved in a primary substrate binding with a putative exosite.

The Michaelis–Menten curves of PARL-mediated cleavage of IQ-PGAM5^20–29^ and IQ-Smac/Diablo^51–60^ were obtained in the presence of different concentrations of PINK1^89–111^. The data sets were fitted globally to competitive, noncompetitive, and mixed inhibition with strong preference for competitive inhibition for both substrates and global R^2^ of 0.93 and 0.90 for IQ-PGAM5^20–29^ and IQ-Smac/Diablo^51–60,^ respectively. The determined K_d_ for IQ-PINK1^89–111^ (represented by K_i_ of the PARL-PINK^89–111^ complex) was 2.4 ± 0.6 μM when IQ-PGAM5^20–29^ was used as the main substrate and 2.3 ± 0.7 μM when IQ-Smac/Diablo^51–60^ was used (53). These competitive inhibition parameters between the two substrates revealed that the assessed PARL substrates bind to the same binding site with similar affinities and are exclusive to each other. These results suggest that the inverse regulation of PINK1, PGAM5, and Smac/Diablo cleavage observed in cells is controlled by other mechanisms such as compartmentalization, involvement of protein partners for substrate presentation or different accessibility of the scissile bond in response to different membrane conditions.

### PARL has bulky substrate specificity preferences distinct from bacterial rhomboid proteases

**Figure 5:**
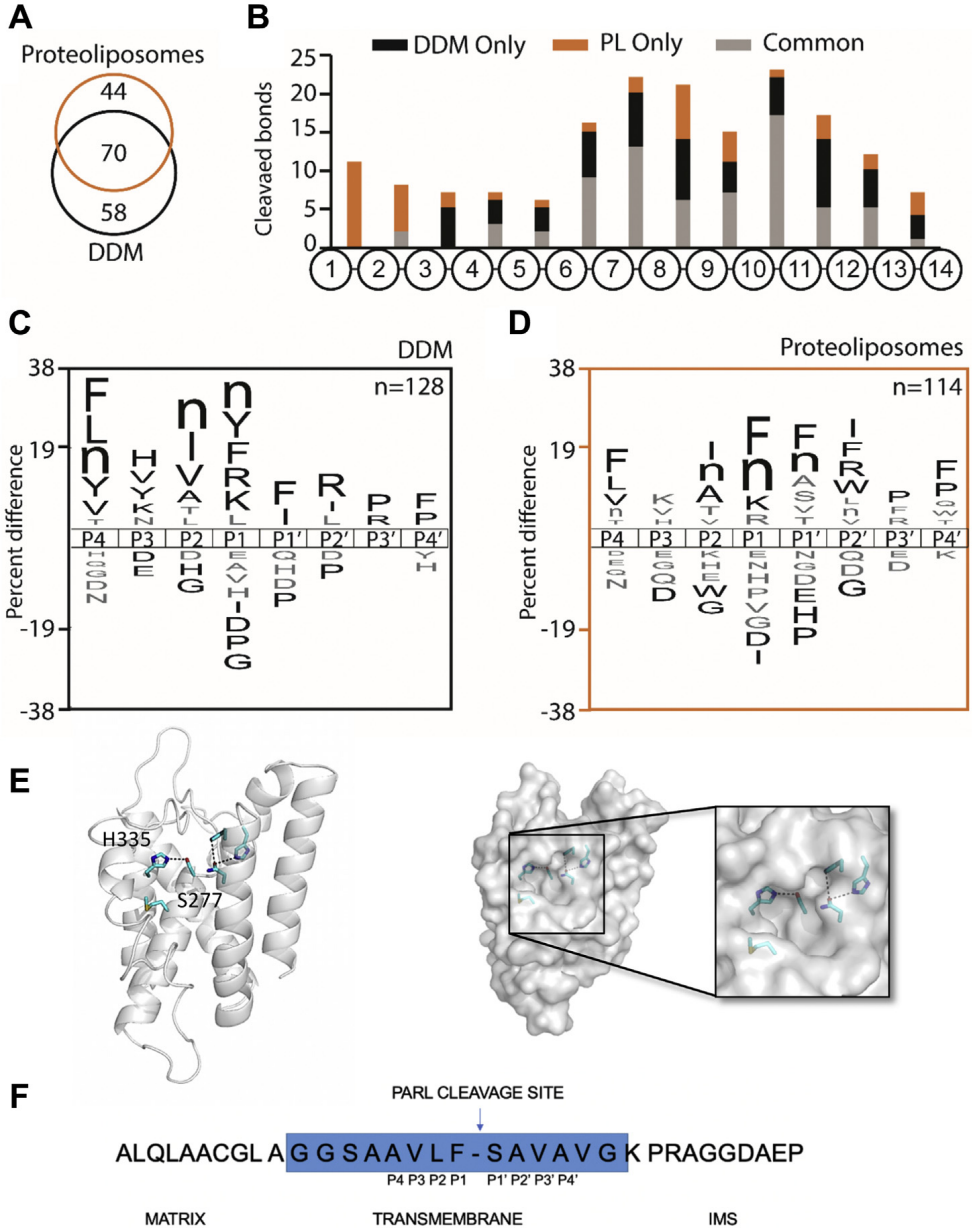
**PARL protease has substrate specificity distinct from bacterial rhomboid proteases.** Substrate specificity parameters using multiplex LC-MS/MS screening with 283 substrates. Samples assessed were PARLΔ77 in DDM with CL added and PARLΔ77 in PL containing PC, PE, and CL. *A*, common peptides cleaved between PARLΔ77 in DDM (*black line*) and PL-reconstituted PARLΔ77 (*orange line*). *B*, position of cleavage of IQ-peptide between PARLΔ77 in DDM and PL. *C*, amino acid preferences for PARLΔ77 in DDM *versus* (*D*) PARLΔ77 in PL. *E*, PARL (catalytic core) homology model based on the HiGlpG structure (2NR9.pdb) reveals a helical bundle and catalytic Ser277-His355 dyad. A surface representation reveals a P1 pocket near the catalytic serine. *F*, N-terminal sequencing of the C-terminal MBP-PGAM5 cleavage product reveals a Phe residue in the P1 position. *Blue* represents the predicted TM boundary of PGAM5.

Bacterial rhomboid proteases are known to cleave substrates with a certain specificity for small side chain residues in the P1 position and bulky hydrophobic residues in the P4 position (23). Analysis of the C-terminal cleavage sites of PARL-generated cleavage products isolated from cell extracts by Edman degradation (8, 39) or mass spectrometry (11) so far revealed no consensus. Using recombinant PARL, we now assessed the cleavage site specificity using a library of 228 synthetic peptides that are each 14 amino acids in length. This library was developed to have all pairwise combinations of neighbor and near-neighbor amino acids. We have previously confirmed that many peptide substrates of this library are cleaved by the bacterial rhomboids from *Providencia stuartii* and *Haemophilus influenza* (36). PARL in buffer containing DDM or reconstituted in proteoliposomes was incubated with an equimolar mixture of peptides and 70 common cleavage products by the enzyme under these two different assay conditions were identified (Fig. 5). We analyzed the location of cleavage within the peptide substrates and discovered that when PARL was assayed in proteoliposomes, it cleaved many peptides near the amino terminus, while PARL in DDM did not (Fig. 5*B*). These data indicated that the lipid environment for PARL may alter its cleavage preference of the enzyme or prevent access of certain substrates to the active site.

Importantly, small amino acids were not found in the P1 position when PARL was assayed in either DDM or PL. In DDM, PARL preferentially cleaved peptides with norleucine (Nle), Tyr, Phe, Arg, and Lys in the P1 position while Phe and Ile are most frequently found in the P1ʹ position (Fig. 5*C*). In addition, hydrophobic amino acids are found at the P4 and P2 position. When PARL was assayed in PL, Phe and Nle were found most frequently in the P1 and P1ʹ positions (Fig. 5*D*). Lys was also significantly enriched in the P1 position, while hydrophobic amino acids were found in the P4, P2, and P4ʹ positions. When these profiles were compared with the sequences surrounding the putative PINK1 cleavage site, VFLA-FGLG, only P1ʹ-Phe and P4-Val are well tolerated from this sequence. In fact, Ala at P1 and Gly at P2ʹ are disfavored by PARL when assayed in DDM and PL, respectively. However, a GlpG-based homology model of human PARL reveals a putative pocket in the P1 position that could accommodate such a bulky side chain (Fig. 5*E*). The P4 position has a consistent bulky residue similar to that for bacterial rhomboid proteases.

When PARL was incubated with the full TM region of MBP-PGAM5, N-terminal sequencing revealed that cleavage occurred between Phe-23 and Ser-24 (Fig. 5*F*). Interestingly, this cleavage site determined in our *in vitro* assay is different from the site previously determined in tissue culture cells, which for PGAM5 was between Ser-24 and Ala-25 (8). Readout of an *in vitro* assay is more direct than determination of N terminus of cleavage fragments isolated from tissue culture cells, so we suggest that the *in vivo* cleavage fragments may become subject to further trimming by additional proteases. In addition, the newly revealed PGAM5 cleavage site, originally thought to be in the center of the TM domain, topologically, is now placed closer to the matrix-exposed rhomboid active site. Taken together with the results from the peptide library (Fig. 5), the *in vitro* determined PGAM5 cleavage sites support the preference for PARL to cleave proteins and peptides with a bulky amino acid such as Phe in the P1 position. This is the first substrate specificity study of PARL, which shows an interesting preference for Phe in the P1 position, which is distinct from most bacterial rhomboid proteases that prefer small side chain residues in the P1 position (23).

## Discussion

In this study, we provide multiple lines of evidence that recombinantly produced human PARL protease is active in both detergent and lipid environments, allowing for a significant advance in our understanding of the regulation of PARL-mediated catalysis. We established a FRET-based assay to monitor proteolytic activity of PARLΔ55 and PARLΔ77 in a continuous manner, which allowed us to gather catalytic parameters of cleavage of three unique substrates, PGAM5, PINK1, and Smac/Diablo, by each PARL construct. Taken together, this is the formal proof that PARL is indeed able to cleave substrates that have been previously identified in cellular assays. When comparing the catalytic parameters, we revealed that the cleavage rates for three known PARL substrates are different with PGAM5 being preferred. We consistently observe that the K_M_ value is the lowest for PGAM5, indicating a higher affinity with the greatest catalytic efficiency. This suggests that substrate specificity or substrate preference might have a role in regulating PARL-catalyzed cleavage.

Overall PARL exhibited a very slow catalytic rate for proteolytic reactions. The bacterial rhomboid proteases from *Haemophilus influenzae* (HiGlpG) and *P. stuartii* (AarA) cleave at a rate of roughly two per minute for their preferred substrates in DDM, while the *E. coli* rhomboid protease GlpG cleaves slowly in DDM, much like PARL, at a rate of approximately six per hour and with a rate of 2.5 min^−1^ in PLs (26, 34, 35). Studies on other intramembrane proteases also suggest that these slow turnover rates are common for intramembrane proteolytic assays performed *in vitro*; intramembrane aspartyl proteases have been found to cleave a physiological FRET peptide substrate at a rate of approximately two per hour, which is not considerably different than what we see for PARL (26, 34, 35, 38). Regardless of the slow rate of substrate turnover, the catalytic parameters are still able to provide valuable information regarding the unique enzyme–substrate interactions for each substrate assessed.

With the recombinant protein and established activity assays in hand, we were able to conduct the competition binding studies to determine if the active site of PARL is the only molecular determinant regulating the substrate selection or if allosteric interactions are involved in the mechanism of cleavage of multiple substrates. The fact that we see competitive inhibition between all three substrates demonstrates that they are binding to the same binding sites on PARL molecule and the existence of allosteric sites is not supported by our data.

Processing of PARL to either its mature Δ53 form or the further truncated Δ77 form has been proposed to be a modulator of protease enzymatic activity. Cellular studies have provided conflicting evidence: an impaired PARL activity is observed when mutation at Ser77 prevents β-cleavage to the PARLΔ77 form, though the PARLΔ53 form appears to be more active toward the PINK1 substrate (7, 21). Using recombinant PARLΔ55 and PARLΔ77, we assessed the cleavage of three unique peptide substrates, IQ-PGAM5, IQ-PINK1, and IQ-Smac. We validated that PARL is catalytically active in either form, thus indicating that processing to the Δ77 form is not required for its proteolytic activity or functionality as was once speculated (7). While these truncations do not serve as an activation switch for the protease, we found that there are measurable differences in the catalytic parameters of cleavage between the two forms. This suggests that PARL truncations identified *in vivo* might regulate aspects of their activity. PARLΔ55 demonstrated significantly lower substrate turnover for IQ-PGAM5 and IQ-PINK1. PGAM5 is known to be preferentially cleaved by PARL upon mitochondrial depolarization, when enhanced β-cleavage and PARLΔ77 formation is also observed (8, 21). Previous studies suggest that PARLΔ77 is catalytically less active toward PINK1 than the longer form of the protease (21), though our data suggest otherwise. This contradictory speculation could be explained by the fact that these cellular studies were performed during times of mitochondrial stress, in which PARLΔ77 formation is enhanced, but PINK1 import to the IMM is impaired, therefore even if PARLΔ77 is more active toward PINK1, it does not have access to the substrate. Interestingly, with the IQ-Smac/Diablo peptide, no significant difference was noticed in any of the catalytic parameters when cleaved by PARLΔ55 or PARLΔ77 in detergent, but we see an increase in proteolysis with PARLΔ77 form in PLs.

While we were able to compare the cleavage of substrates mediated by either PARLΔ55 or PARLΔ77, many questions remain unanswered in regard to these forms of PARL. It is important to note that varying amounts of PARL truncations are detected in different tissues, suggesting that β-processing and even function of specific PARL forms could be tissue-specific (20).

This raises questions on the role of the roughly 20 amino acid N-terminal region of protease molecule that is removed upon β-cleavage. We can make several speculations on its potential function, which may include protein stabilization or aiding in substrate recognition. Based on its localization to the matrix side of the IMM, it may be involved in mediating interactions with proteins that reside in the mitochondrial matrix. PARL is a member of a larger proteolytic hub in the IMM consisting of PARL protease, the i-AAA protease YME1L, and the scaffold stromatin-like protein 2 (51); however, it is still unknown what form of PARL associates with the complex. Plausibly, N-terminal region is required for protein–protein interactions between PARL and the SLP2 scaffold protein or truncation of PARL may alter associations with YME1L.

We also demonstrated that cardiolipin has a significant effect on the activity of PARL, which presents the first evidence that lipids may modulate the activity of the mitochondrial rhomboid protease. Cardiolipin is the lipid exclusive to the IMM of eukaryotic cells, the membrane in which PARL is localized. In the IMM, CL represents approximately 10% of the total lipid, which is known to be essential to the activity of numerous IMM proteins (54). The finding that CL can influence the proteolytic activity of PARL is not overly surprising as there is considerable evidence that the activity of rhomboids can be modulated by lipids and that proteins of the IMM are influenced by the presence of CL. We determined that a 25:1 M ratio of CL to PARL results in the greatest increase in proteolytic activity compared with the no-lipid condition. Such increase in activity may be explained either by enhancement of protease stability or by CL binding to a specific site on PARL molecule, thereby inducing subtle conformational changes that facilitate substrate binding or substrate entrance to the active site. There are currently over 60 different proteins, many from the mitochondria, reported to interact with CL, and for over 20 of these high-resolution structures have been determined with at least one CL molecule present (55). It is worth noting that CL is often seen as an interactor within protein complexes in the IMM, exemplified by its critical role in both stability and function of the respiratory supercomplexes; there are predicted to be 200–400 cardiolipin molecules associated with the respiratory supercomplexes from bovine heart (55). Given the fact that PARL is thought to interact with YME1L-SLP2 complex within the IMM, CL might facilitate the formation, stability, and organization of such a complex.

Characterization of PARL protease revealed a unique substrate specificity different from most bacterial rhomboid proteases. The preference for a large hydrophobic residue, particularly Phe, in the P1 position is in stark contrast to the substrate specificity of most bacterial rhomboid proteases, which allow only the small nonpolar residue Ala in the P1 position (23, 56). In fact, no cleavage of the TatA substrate occurs by *P. stuartii* rhomboid protease AarA when Phe is mutated into the P1 position of the substrate cleavage site (23). Analysis of the *E. coli* rhomboid protease GlpG with a peptide substrate transition analog revealed a similar preference (56). Thus far, YqgP from *B. subtilis* is the only bacterial rhomboid protease known to cleave with Phe at the P1 position (23). YqgP is evolutionarily distinct from the *E. coli* GlpG (18), which suggests evolutionary pressure on substrate specificity. However, we still see that a hydrophobic Phe residue is conserved in the P4 position between bacterial rhomboids and PARLΔ77. Previous proteomics study identified six PARL substrates with three having an Ala, while the others either a Ser or Cys residue in the P1 position (11). The contradiction with our data could be explained by the fact that in the previous report lysates of HEK293 cells were used as opposed to purified protease for substrate identification. Structural modeling of PARL also supports its preference for a bulky amino acid at P1 position. When looking at the surface representation of a homology model of PARL based on the bacterial rhomboid protease HiGlpG structure, a large substrate binding pocket that can easily facilitate the entrance of a bulky residue, such as a Phe, is observed within the catalytic core of the enzyme (Fig. 5*E*). We see that negatively charged amino acids are highly unfavorable within the P4 to P4’ positions; this is likely due to disruption of the oxyanion hole that would result from a negative charge entering into the catalytic core of the enzyme (57).

The substrate specificity profile for PARL also suggests that the enzyme has overall broad substrate specificity toward TM substrates based on the preference for residues such as Phe, Ala, Val, Ile, and Pro, which are commonly associated with TM regions of a protein. Furthermore, in the region directly C-terminal to the cleavage site, there is a preference for the helix-destabilizing or helix-breaking residues Pro and Gly (6), which supports the evidence gathered for bacterial rhomboids suggesting that helix-destabilizing residues are required to facilitate unwinding of the helical TM substrate segment for better access to the cleavage site (58). It also indicates that there are likely other factors that regulate intramembrane proteolysis, rather than a highly specific substrate recognition motif. The broad substrate specificity obtained for PARL supports previous work on the yeast mitochondrial rhomboid that demonstrated large sequence variability in cleavable substrates (59). Other intramembrane proteases, such as γ-secretase, have also been established to have broad specificity, with γ-secretase sometimes being referred to as the “proteasome of the membrane” with over 100 identified substrates (60, 61). Most likely for mitochondrial rhomboids, there are also numerous substrates that have yet to be identified.

Our study characterized several aspects of PARL-mediated cleavage that were addressed by using *in vitro* proteolytic assays with a recombinant enzyme. We established activity assays with specific FRET-peptides, which could be used for downstream applications such as inhibitor screening. These assays confirmed that our recombinant protease retained activity after purification and determined the catalytic parameters of cleavage of three main substrates. Our *in vitro* studies present a significant advancement in the field as the majority of previous kinetic studies on rhomboid proteases have been limited to the bacterial rhomboid proteases and provide new methods for characterizing regulatory and mechanistic aspects of PARL’s proteolytic functions.

## Experimental procedures

### Expression and purification of recombinant PARL

PARL gene (PARLΔ77, PARLΔ77-S277A, or PARLΔ55) was cloned into pPICZA vector, followed by a TEV cleavage site, with a C-terminal GFP and hexahistidine-tag (22). The S277A mutant was created using site-directed mutagenesis technique (Q5 Site-Directed Mutagenesis Kit, NEB) with GTCATGATGGCACCAGCT GCACCAAGTGATGGT as forward primer and ACCATCACTTGGTGCAGCTGGTGCCATCATGAC as a reverse primer. An identified high-expressing clone was grown overnight at 28 °C in 100 ml of BMGY media to an OD_600_ of 4. A total of 6 L of BMGY media was subinoculated to a starting OD_600_ of 0.03 and grown for 20 h at 28 °C. Cells were harvested by centrifugation and cell pellets were resuspended in an equal volume of BMMY induction media. Cultures were induced for 48 h at 24 °C, with fresh methanol being added after 24 h to a final concentration of 1% (v/v). Cells were harvested and resuspended in TBS buffer (50 mM Tris-HCl pH 8.0, 150 mM NaCl). Cells were resuspended in TBS with PMSF and lysed by passage through a Constant Systems cell disruptor at 38.2 kPSI, and membranes were isolated by ultracentrifugation. Membranes were homogenized in 50 mM Tris-HCl pH 8.0, 200 mM NaCl, 5% glycerol, 20 mM imidazole, 1 mM PMSF, and solubilized using 1.2% Triton X-100. Insoluble material was pelleted by ultracentrifugation and the supernatant bound to HisPur cobalt resin (Thermo Fisher) by gravity flow-through column. The protein-bound resin was washed with 10 mM imidazole and eluted with imidazole (50 mM Tris-HCl pH 8.0, 300 mM NaCl, 20% glycerol, 0.1% DDM [Anatrace], 1 M imidazole). The purified PARL-GFP fusion protein was digested by incubation with TEV protease and 1 mM TCEP overnight at 4 °C. Dialysis was performed for 2 h to remove imidazole and TCEP (50 mM Tris-HCl pH 8.0, 300 mM NaCl, 20% glycerol). PARL was purified from GFP and TEV using HisPur Ni-NTA agarose resin (Thermo Fisher). Flow-through was collected and concentrated using a 10000 MWCO concentrator (Millipore). Protein concentration was determined by BCA assay (Pierce BCA Protein Assay Kit, ThermoFisher). Purified protein, at a concentration of 1 mg/ml, was incubated on ice with dried cardiolipin (Sigma-Aldrich) with protein: cardiolipin molar ratio of 25:1 Protein–lipid sample was aliquoted, flash-frozen, and stored at –80 °C. After each purification, the quality of purified PARL was controlled by measuring its enzymatic activity.

### FRET-PINK1 purification

Residues 70–134 of Human PINK1-WT were cloned into the pBad/HisB vector that already encoded for the engineered FRET pair, CyPet and YPet (derived from cyan fluorescence protein and yellow florescence proteins); this new pair exhibited 20-fold energy transfer efficiency when compared with the parental pair (62). The vector was transformed into TOP10 chemically competent *E. coli* cells (Thermo Fisher). Transformed cells were grown overnight at 37 °C on LB agar plates containing 100 μg/ml ampicillin. One transformant colony was selected and grown overnight at 37 °C in 120 ml of LB medium containing 100 μg/ml ampicillin. A total of 6 L LB media was subinoculated with 20 ml of overnight culture and grown to an OD_600_ of 0.7 at 37 °C. Cultures were induced by addition of 0.02% (v/v) L-arabinose for 8 h at 24 °C. After induction, cells were harvested by centrifugation in a Beckman JLA8.1000 rotor (6900*g*, 20 min, 4 °C), flash-frozen in liquid nitrogen, and stored at –80 °C. Harvested cells were thawed on ice and resuspended in a 4:1 buffer volume to cell pellet weight ratio in resuspension buffer (50 mM Tris-HCl pH 8.0, 500 mM NaCl, 20% glycerol, 10 μg/ml DNase, 1 mM PMSF, two EDTA-free protease inhibitor cocktail tablets). Resuspended cells were lysed using an Emulsiflex with a maximum pressure of 40 kPSI. Following cell lysis, the lysate was subjected to centrifugation using a Beckman TI45 rotor (31,300*g*, 20 min, 4°C) to pellet cell debris and unlysed cells. The supernatant was incubated with 1% (v/v) Triton X-100 at 4°C for 30 min with stirring. Supernatant was then passed through 1 ml settled HisPur cobalt resin (ThermoFisher) by gravity flow to allow binding of FRET- PINK1-His to the resin. Protein was eluted (50 mM Tris-HCl pH 8.0, 500 mM NaCl, 20% glycerol, 250 mM imidazole), pooled, and concentrated for loading onto the Superdex 200 column for size-exclusion chromatography. Size-exclusion chromatography fractions were analyzed by SDS-PAGE and fractions containing FRET-PINK1 protein were pooled and concentrated. Concentrated sample was aliquoted, flash-frozen with liquid nitrogen, and purified HsFRET-PINK1(70–134) was stored at –80 °C for subsequent use.

### PINK1 TM expression and purification

The sequence of PINK1 TM domain (amino acids 89–111) was codon optimized for *E. coli* expression and cloned into pMAL-c2 vector (New England Biolab) with N-terminal Maltose Binding Protein (MBP) followed by a tobacco etch virus (TEV) cleavage site. The vector was transformed into DH5α cells and the protein was induced with 0.5 mM IPTG and expressed for 3 days at 24 °C. Cells were harvested, resuspended in 20 mM KPO_4_ (pH 8), 120 mM NaCl, 50 mM glycerol, 1 mM EDTA, 1 mM PMSF, 1 mM DTT, and lysed using an Emulsiflex with a maximum pressure of 40 kPSI. 0.5% Triton X-100 was added postlysis and cell debris was removed by centrifugation at 40,000*g* for 30 min at 4 °C. The supernatant was loaded onto amylose resin (Amylose Resin High Flow, NEB), equilibrated with 20 mM KPO_4_, pH 8.0, 120 mM NaCl, 1 mM EDTA buffer, and the protein was eluted with 40 mM maltose in equilibration buffer. MBP tag was cleaved off by MBP-PINK1 incubation with recombinant TEV protease (1.5 mg of TEV per 30 mg of fusion protein) at 16 °C for 4 to 8 days. To extract PINK1 TM segment 1/6 of the sample volume of 60% w/v trichloroacetic acid (TCA) was added to protein mixture and incubated for 30 min on ice. The precipitate was pelleted for 10 min at 10,000*g*, rinsed three times with ddH_2_O, resuspended in 50:50 isopropanol: chloroform, and mixed with a homogenizer. To this mixture, 1–2 ml of ddH_2_O was added into each tube and incubated overnight allowing for separation of the organic and aqueous layers. The organic layer was transferred into a clean tube and fresh 1–2 ml of was aliquoted into a sample and left overnight at room temperature. This separation was repeated until all white precipitate was removed and organic phase was considered clean. Organic layers were combined and dried down under nitrogen or argon gas. The PINK1 peptide was resuspended in ∼6–8 ml of 7 M guanidine-HCl, 50 mM KPO_4_ buffer (pH 8) and injected onto an Agilant Zorbax SB-300 C8 silica-based, stainless steel 25 cm × 1 cm column, which was preheated to 60 °C. The column ran at 60 °C with a flow rate of 1 ml/min. An isopropanol gradient (20%–80%) against 0.05% TFA/water was used to elute the protein. PINK1 TM typically eluted at ∼50% isopropanol. Determination of fractions containing the peptide was established by running 6% urea gels, which were visualized through silver staining.

### MBP-PGAM5 expression and purification

The sequence of the PGAM5 TM region (amino acids 1–46) was cloned into *E. coli* expression vector pET-25b(+) (Novagen) with N-terminal MBP and C-terminal Thioredoxin 1 followed by a triple FLAG-tag and a C-terminal hexahistidine-tag. The vector was transformed into chemical competent Rosetta 2 (DE3) cells (Novagen), grown in LB medium. Expression of the protein was induced with 0.3 mM IPTG and expressed for 2 h at 37 °C. Cells were harvested by centrifugation at 3500 rpm for 15 min at 4 °C and resuspended in 20 mM HEPES pH 7.4, 150 mM NaCl, 5 mM MgCl_2_, 10% glycerol, 1 mM PMSF, 5 mM β-mercaptoethanol. Prior to lysis, 200 μg/ml lysozyme, 1 mM PMSF, and benzonase (2.5 ku, Merck Millipore) were added and cells were lysed using Emulsiflex (Avestin) with a maximum pressure of 15 kPSI (100 MPa). Crude membranes were obtained by ultracentrifugation at 29,000 rpm for 45 min at 4 °C. The membrane pellet was resuspended in 50 mM HEPES pH 7.4, 150 mM NaCl, 5 mM MgCl_2_, 10% glycerol, 1 mM PMSF, 5 mM β-mercaptoethanol. MBP-PGAM5 was solubilized from the crude membranes with 1.5% DDM for 1 h on a rotating wheel at room temperature. Extraction of MBP-PGAM5 from membrane debris was done by ultracentrifugation at 29,000 rpm for 1 h at 4 °C. Cleared extract was batch incubated with Ni-NTA beads (Macherey-Nagel) for 1 h on a rotating wheel at room temperature for His-tag affinity purification. Bound MBP-PGAM5 was washed with 50 mM HEPES pH 7.4, 300 mM NaCl, 10% glycerol, 50 mM imidazole, 0.05% DDM and eluted with 50 mM HEPES pH 7.4, 300 mM NaCl, 10% glycerol, 400 mM imidazole, 0.05% DDM. Determination of fractions containing the peptide was established by SDS-PAGE running 12% acrylamide gels, which were visualized through Coomassie staining.

### MBP-PGAM5 cleavage assay

Five micrograms (4 μM) of *E. coli* purified MBP-PGAM5 was incubated with either 0.44 μg (0.7 μM) PARL or 0.44 μg (0.7 μM) catalytic inactive PARL-S277A purified from *P. pastoris* for 1.5 h at 30 °C in cleavage buffer containing 50 mM Tris pH 8.0, 150 mM NaCl, 10% glycerol, 0.3% DDM. Determination of peptide cleavage was established by SDS-PAGE using 12% acrylamide gels, which were visualized through Coomassie staining.

### N-terminal sequencing by Edman degradation

In total, 8–16 μg of *E. coli* purified MBP-PGAM5 was incubated with 0.4 μg of *P. pastoris* purified PARL for 2 h at 37 °C in cleavage buffer containing 50 mM Tris pH 8.0, 150 mM NaCl, 10% glycerol, 0.3% DDM. Protein fragments were separated by SDS-PAGE running 12% acrylamide gels and transferred to a PVDF membrane by wet blot (glycine buffer) for 1 h at 100 V. Protein fragments were stained with Coomassie overnight and the C-terminal fragment (CTF) was then analyzed in four cycles by Edman degradation (TOPLAB).

### FRET-based protease kinetic assay

Assays with FRET-PINK1^70–134^ were conducted as previously described (26). For EDANS/Dabcyl 10-mer IQ peptides (PINK1, PGAM5, Smac/Diablo), lyophilized peptides were initially dissolved in DMSO to obtain a stock solution. The IQ peptide substrates in a concentration range of 0.1–70 μM were incubated with activity assay buffer (50 mM Tris-HCl pH 7.0, 150 mM NaCl, 10% glycerol, 0.1% DDM) in a 384-well black-bottomed plate at 37 °C for 30 min in a multiwell plate reader (SynergyMx, BioTek). For all concentrations of IQ peptide, the DMSO was kept constant at 5%. Following preincubation, PARL was added to a final concentration of 0.8 μM to initiate the cleavage reaction. Fluorescence readings were taken every 3 min over a 3 h time course at ƛ_ex_ = 336 nm and ƛ_em_ = 490 nm. The initial velocity was determined from the fluorescence readings over the time course. For each substrate concentration, a no-enzyme control was subtracted to eliminate background fluorescence changes not related to substrate cleavage. Relative fluorescence units were converted to concentration (μM) by determining the maximum change in fluorescence observed for each substrate concentration when fully digested. GraphPad Prism software was used for Michaelis–Menten analysis of kinetic curves. All kinetic data were obtained using at least three biological replicates (different enzyme preparations) with technical duplicates for each experiment. For kinetic measurements in detergent with all three substrates, the activity assays were repeated at least five times. In addition, after each protease purification, the quality control of purified PARL was controlled by measuring its enzymatic activity. Minimum of three experimental replicates with two technical replicates were used for data analysis.

### Reconstitution in proteoliposomes

*E. coli* polar lipids (Avanti), 400 μg in chloroform, were dried under nitrogen stream in a glass tube to yield a thin film of lipid. The tube was incubated overnight in a desiccator to completely remove all traces of solvent. In total, 50 μl of water and DDM detergent was added to the lipid film for resuspension at room temperature for 10 min, followed by the addition of purified PARL (400 μg) in 50 mM Tris-HCl, pH 8.0, 150 mM NaCl, 20% glycerol, 0.1% DDM, to yield the final weight ratio of 1 PARL:1 lipid:2 detergent. The detergent was slowly removed by the addition of SM2 Biobeads (Bio-Rad) while stirring on ice for 6 h to allow for the generation of proteoliposomes (PL); the process was controlled by the regimen of Biobead addition. To purify the PL, 50%:20% sucrose density gradient ultracentrifugation was used. A TAMRA probe (ThermoFisher) was used to determine the orientation of reconstituted PARL, which specifically and covalently labels serine residues of an enzymatically active serine protease. In total, 2 μM of TAMRA probe was added to PL samples and incubated for 1 h, allowing the TAMRA probe to label only the outward-facing accessible active sites. The same amount of PL, but with 1% DDM added to dissolve lipid vesicles incubated with 2 μM of TAMRA probe at the same conditions, was used as a benchmark for 100% accessible protein amount. The reaction was quenched with addition of SDS-containing sample buffer and the protein samples were visualized with SDS-PAGE followed by fluorescent gel scanning. The bands were quantified by densitometry analysis. The difference in labeling between the first and the second samples gave us the proportion of accessible protein in PLs *versus* the whole amount of protein. Coomassie staining was used to normalize the amount of protein loaded.

### Activity assay in proteoliposomes

The activity assay with PARL reconstituted in PL was performed the same way as for PARL in DDM with the only difference being the activity buffer, where DDM was omitted (50 mM Tris-HCl pH 7.0, 150 mM NaCl, 10% glycerol). For inhibitory studies, PARL in PL (0.8 μM) was incubated with inhibitors (20 μM) in activity buffer for 30 min, and then the proteolytic reaction was started with the addition of substrate (5 μM).

### Molecular modeling

Human PARL, isoform 1, was modeled using iTASSER with HiGlpG as the template (63), without any additional restraints (63). Residues 1–167 were removed from the modeling due to low homology with bacterial rhomboid protease crystal structures.

### Multiplex substrate profiling by mass spectrometry

Multiplex substrate profiling by mass spectrometry (MSP-MS) assays was performed in quadruplicate. In total, 1 μM of PARLΔ77 was incubated with an equimolar mixture of 228 synthetic tetradecapeptides at a final concentration of 0.5 μM for each peptide in 50 mM Tris-HCl pH 7.0, 150 mM NaCl, 10% glycerol, with or without 0.1% DDM. The sequence of each peptide is listed in Supplementary Data File. These peptides have been validated as substrates for a wide variety of proteolytic enzymes (PMID 23023596, 24073241, 25944934) including bacterial rhomboid proteases (30705125). For each assay, 20 μl of the reaction mixture was removed after 0, 60, and 240 min of incubation. Enzyme activity was quenched by adding GuHCl (MP Biomedicals) to a final concentration of 6.4 M, and samples were immediately stored at –80 °C. All samples were desalted using C18 spin columns and dried by vacuum centrifugation.

Approximately 2 μg of peptides was injected into a Q-Exactive Mass Spectrometer (Thermo) equipped with an Ultimate 3000 HPLC. Peptides were separated by reverse-phase chromatography on a C18 column (1.7 μm bead size, 75 μm × 25 cm, 65 °C) at a flow rate of 300 nl/min using a 60-min linear gradient from 5% to 30% B, with solvent A: 0.1% formic acid in water and solvent B: 0.1% formic acid in acetonitrile. Survey scans were recorded over a 150–2000 m/z range (70,000 resolutions at 200 m/z, AGC target 3 × 10^6^, 100 ms maximum). MS/MS was performed in data-dependent acquisition mode with HCD fragmentation (28 normalized collision energy) on the 12 most intense precursor ions (17,500 resolutions at 200 m/z, AGC target 1 × 10^5^, 50 ms maximum, dynamic exclusion 20 s).

Data was processed using PEAKS 8.5 (Bioinformatics Solutions Inc). MS^2^ data were searched against the 228 tetradecapeptide library sequences with decoy sequences in reverse order. A precursor tolerance of 20 ppm and 0.01 Da for MS^2^ fragments was defined. No protease digestion and modification were specified. Data were filtered to 1% peptide-level false discovery rates with the target-decoy strategy. Peptides were quantified with label-free quantification and data are normalized by medians and filtered by 0.3 peptide quality. Missing and zero values are imputed with random normally distributed numbers in the range of the average of smallest 5% of the data ± SD. Cleaved sequences were defined as peptide products that increase by a fold change of >8 and q value <0.05 (by Student *t* test) between 0 min and 240 min. IceLogo software was used for visualization of amino-acid frequency surrounding the cleavage sites. Amino acids that were most frequently observed (above axis) and least frequently observed (below axis) from P4 to P4ʹ positions were illustrated. Norleucine (Nle) was represented as “n” in the reported profiles. Mass spectrometry data and searching results have been deposited in MassIVE with accession number, MSV000085295.

### Data availability

All data is located in the article. Raw data associated with the mass spectrometry can be found in the supplemental information.

## Supporting information

This article contains supporting information.

## Conflict of interest

The authors declare that they have no conflicts of interest with the contents of this article.
