# Supplementary material for: Insights into the catalytic properties of the mitochondrial rhomboid protease PARL
Source: J Biol Chem. 2021 Feb 6;296:100383. doi: 10.1016/j.jbc.2021.100383 (PMC7966987; doi:10.1016/j.jbc.2021.100383)
Supplement: Figures S1 to S9 and Tables S1 to S8 [file mmc1.pdf]

Supplemental information:
Insights into the catalytic properties of the mitochondrial rhomboid protease PARL

Laine Lysyk[1], Raelynn Brassard[1], Elena Arutyunova[1], Verena Siebert[2], Zhenze Jiang[3], Emmanuella Takyi[1], Melissa Morrison[1], Howard S. Young[1], Marius K. Lemberg[2], Anthony J. O'Donoghue[3], M. Joanne Lemieux[1]*

[1] Department of Biochemistry, Faculty of Medicine and Dentistry, Membrane Protein Disease Research Group, University of Alberta, Edmonton T6G 2R3, Alberta, Canada
[2] Center for Molecular Biology of Heidelberg University (ZMBH), DKFZ-ZMBH Alliance, Im Neuenheimer Feld 282, 69120 Heidelberg, Germany
[3] Skaggs School of Pharmacy and Pharmaceutical Sciences, University of California San Diego, 9500 Gilman Drive, La Jolla, CA 92093, USA

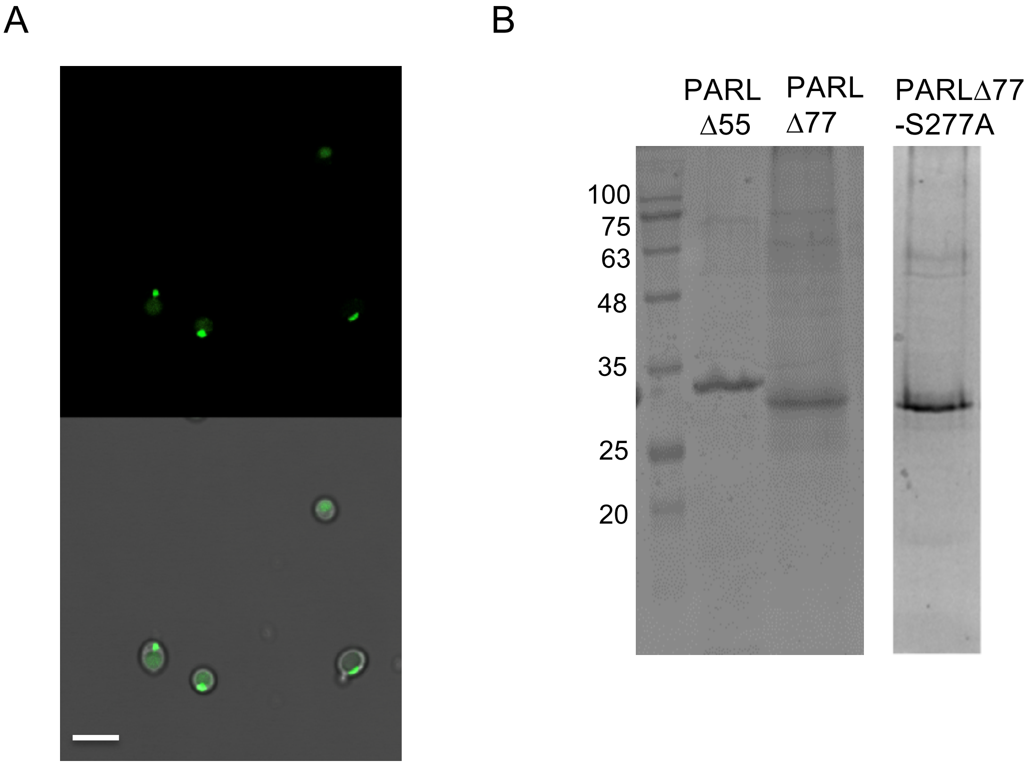

Figure S1. A. PARLΔ77-GFP in *Pichia pastoris* showing the enzyme localizes to the vacuole compartment. The PARL (green) direct fluorescence signal (Top) was merged with a phase-contrast image of the cells (gray). Images were collected on a Zeiss LSM 410 confocal microscope. The scale bar represents 4 μm. **B.** Recombinant human PARL was expressed in *P. pastoris* either truncated at residue Δ55 or Δ77. An inactive PARL-Δ77-S277A mutation was also generated**.**

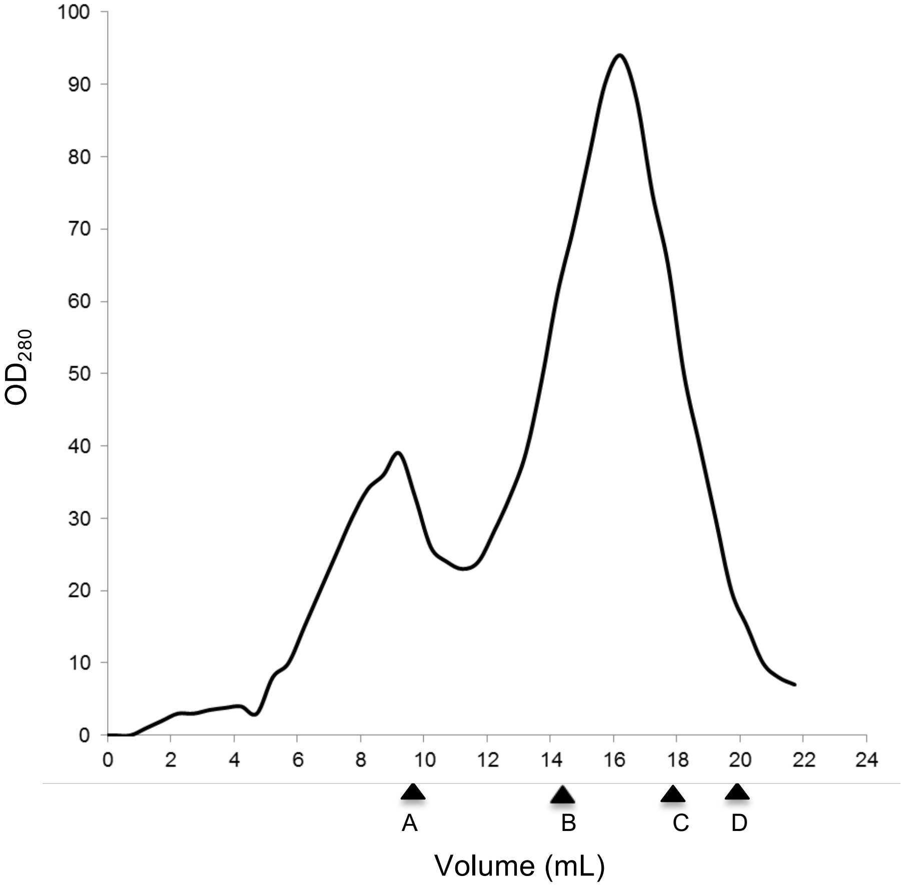

Figure S2. SEC of PARLΔ77 reveals the protein exists in monomeric state in DDM. The protein sample was loaded on a Superdex 200 (10x300) column in 50 mM Tris-HCl, pH 8.0, 5% glycerol, 200 mM NaCl, 0.1% DDM. The column void volume was 8.32 ml. The arrows represent Molecular Weight Standards: **A**-Thyroglobulin (MW, 670 kDa; Stokes radius 85 Å); **B** - γ-Globulin (MW 158 kDa; Stokes radius 52.9 Å); **C** - Ovalbumin (MW 44 kDa; Stokes radius 30.5 Å); **D** - Myoglobin (MW 17 kDa, Stokes radius 20.7 Å).

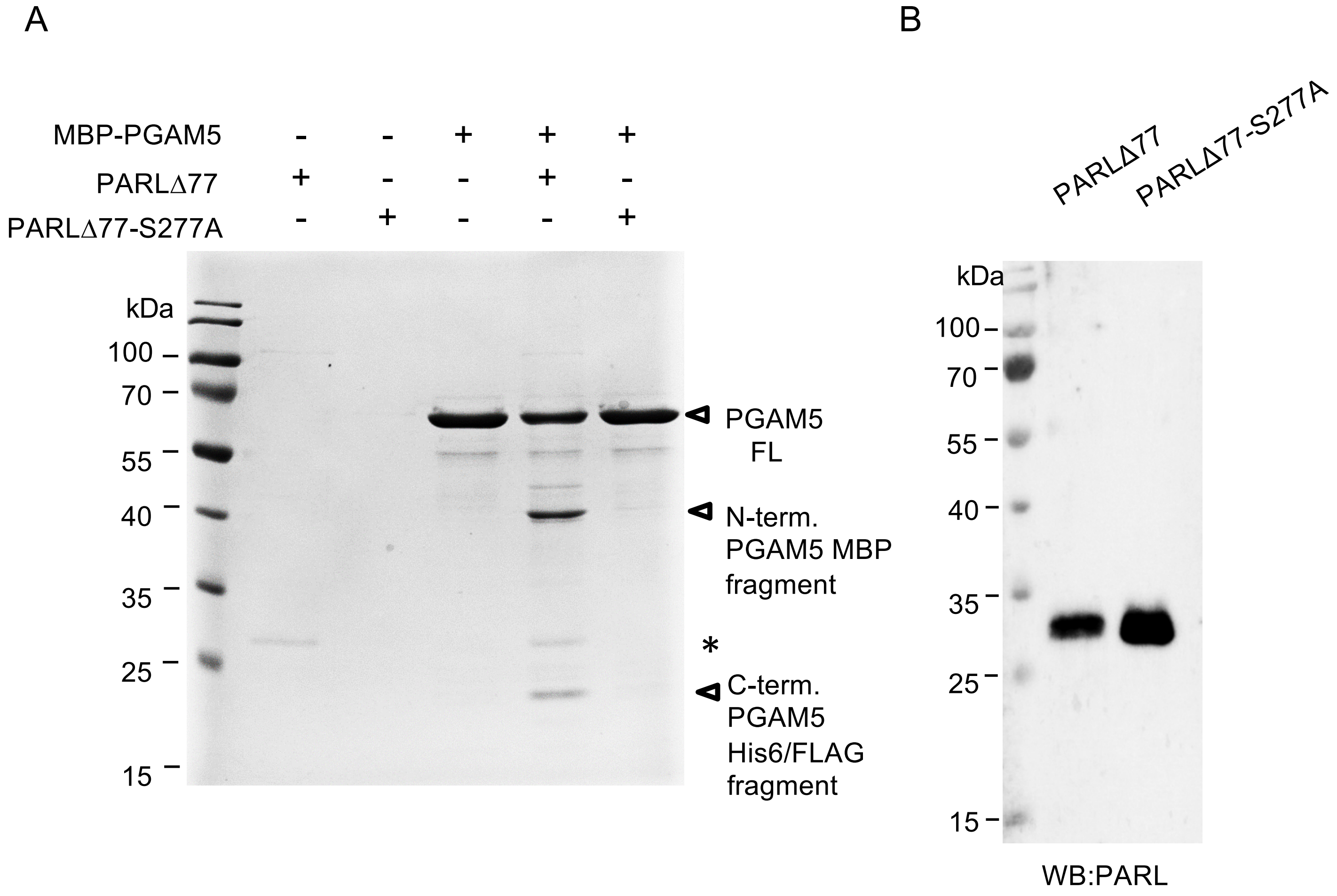

Figure S3. A. Incubation of recombinant PARLΔ77 with MBP-PGAM5 followed by separation on 12% SDS-PAGE reveals N- and C-terminal substrate fragments. The substrate is not cleaved when incubated with inactive PARLΔ77-S277A mutant. *: minor contaminant from PARLΔ77 preparation **B.** Western blot analysis of PARL Δ77 and PARLΔ77-S277A used for MBP-PGAM5 cleavage assay (panel A) reveals the amount of proteases in the assay samples. PARL protease was visualized with anti-PARL antibody (Abcam, USA).

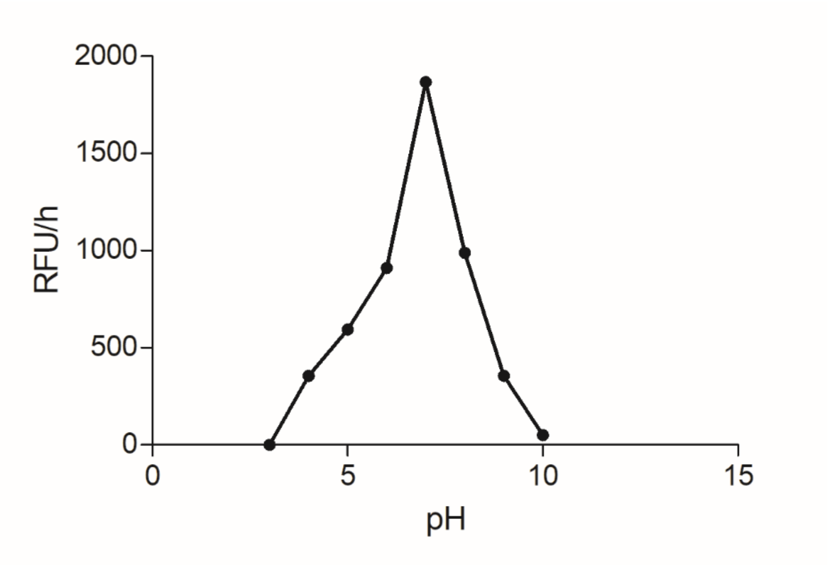

Figure S4. PARLΔ55 activity dependence on pH. The proteolysis reaction was performed at 37°C for 4 h using 5 μM of FRET-PINK1$^{70\text{-}134}$ as substrate and 0.8μM of PARLΔ55 in reaction buffer, at pH interval spanning from 2 to 9 with 50 mM of a broad-range pH triple buffer (boric acid:citric acid:phosphate, (Carmody WR. An easily prepared wide range buffer series. J Chem Educ 40: A386, 1961) with 150 mM NaCl, 20% glycerol, 0.1% DDM.

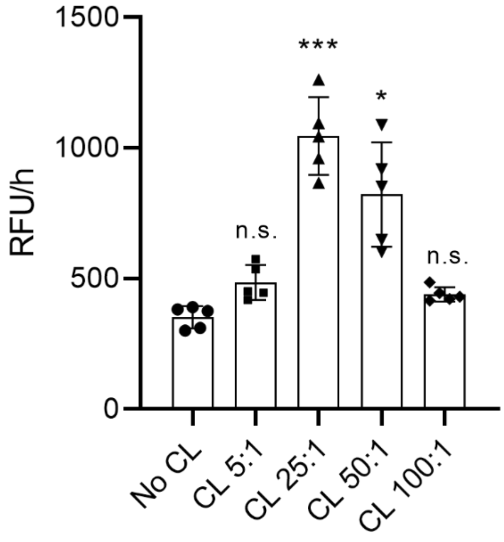

Figure S5. The dependence of PARLΔ77 activity on CL concentration. The initial velocities of PARLΔ77 were measured in the presence of different molar ratios of CL using FRET-PINK1$^{70\text{-}134}$ substrate. One-way ANOVA with multiple comparisons was performed between the no-lipid condition and each molar ratio of CL:protein to determine if differences observed were significant. A total of five experimental replicates performed in duplicate were used for data analysis (N = 5). Individual data points (black dots) are indicated on the bar graphs, representing the mean ±SEM

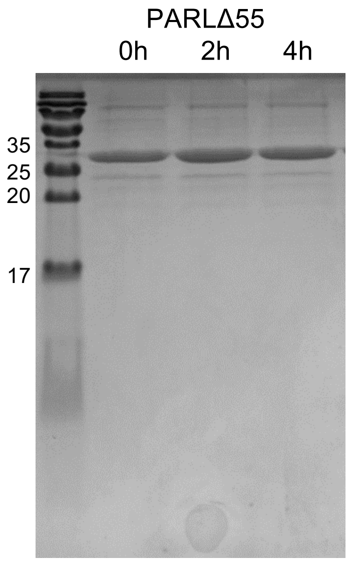

Figure S6. The assessment of PARLΔ55 self-cleavage. The protease was incubated in activity buffer (50 mM Tris-HCl, pH 7.0, 200 mM NaCl, 20% glycerol, 0.1% DDM) at 37°C. The protein samples were taken out after 2 and 4 hours of incubation. No additional bands were observed suggesting that PARLΔ55 preserved integrity at least within the incubation time.

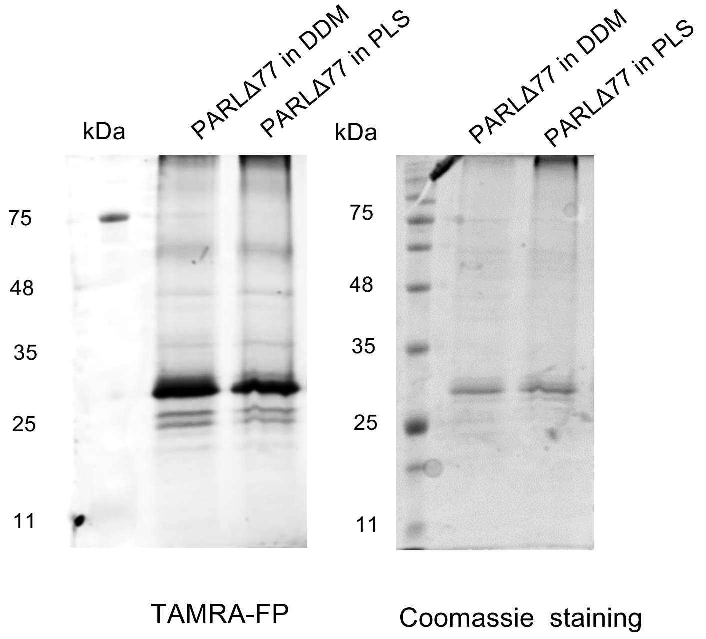

Figure S7. SDS-PAGE of PARLΔ77 with TAMRA probe. To determine the orientation of PARL active site in the proteoliposomes (PL), PARL-PLs sample and PARL-PLs sample incubated with DDM to dissolve PLs were incubated with TAMRA probe. The samples were run on 14% SDS-PAGE and visualized using fluorescent imager. The bands were quantified by densitometry analysis. The percentage of outward-facing PARL molecules in PL was calculated from the difference in labeling between the unsolubilized and solubilized samples (the proportion of accessible protein in PLs versus the whole amount of protein). The amount of protein loaded on the gel was quantified by Coomassie staining of both PARL-PLs samples.

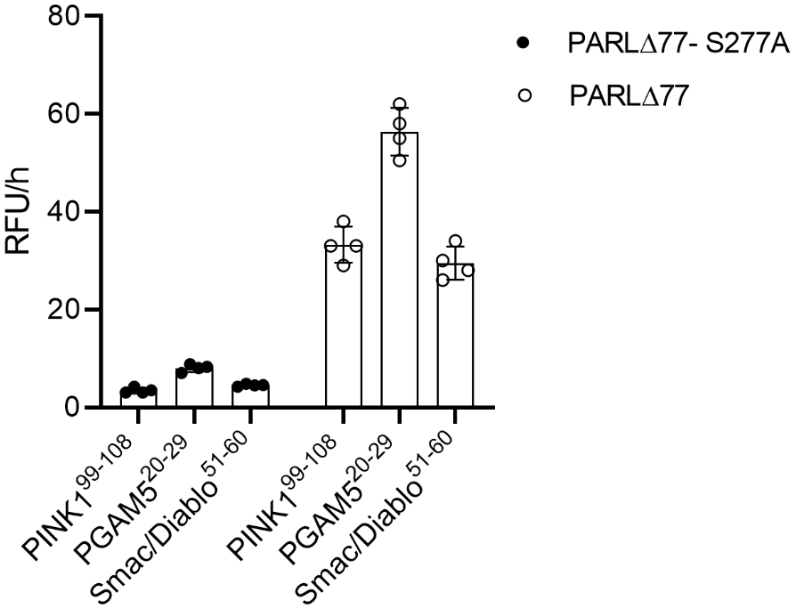

Figure S8. Inactive PARLΔ77-S277A in PLs shows negligible activity with PARL substrates. The activity of PARLΔ77 and PARLΔ77-S277A was measured with 2.3 µM of each substrate. Individual data points (black and open dots) are indicated on the bar graphs, representing the mean (N=4) ± SEM

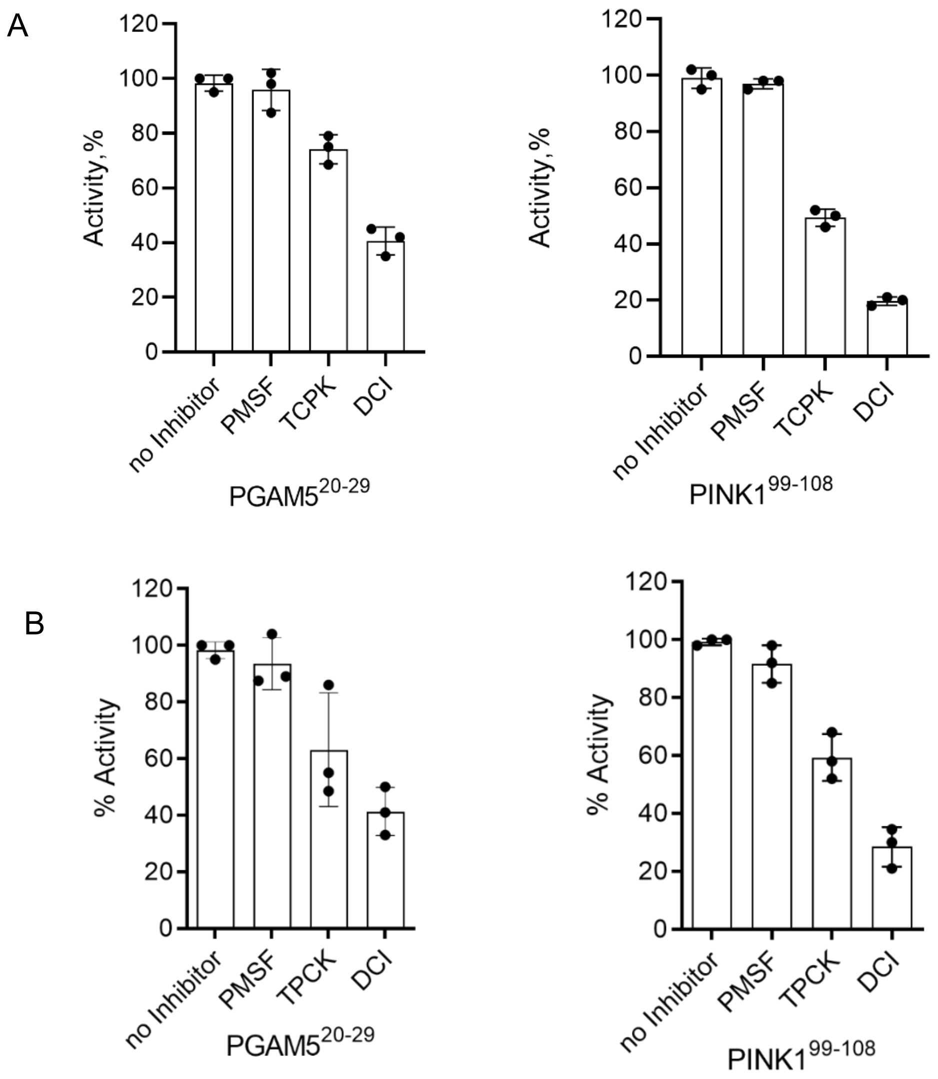

Figure S9. PARL is weakly inhibited by commercially available serine protease inhibitors. Bar graphs for percent activity of detergent solubilized (**A**) and proteoliposome reconstituted (**B**) PARLΔ77 with 100 µM of inhibitor added prior to the addition of IQ-PINK1 and IQ-PGAM5 substrates. Experiments were conducted in duplicate with an N=3. Individual data points (black dots) are indicated on the bar graphs, representing the mean ±SEM.

Supplemental Tables

	PARLΔ55	PARLΔ77	PARLΔ55S77N
K_M (μM)	5±1	3±1	6±2
k_{cat} (h^{-1})	0.43±0.09	0.73±0.06	0.47±0.09
k_{cat}/K_M ($\mu M^{-1} h^{-1}$)	0.09±0.009	0.24±0.03	0.08±0.01

Table S1. Catalytic parameters of PARLΔ55 and PARLΔ77 with FRET-PINK1$^{70\text{-}134}$ substrate.

Peptide name	Peptide sequence
IQ-PINK1$^{99\text{-}108}$	(DABCYL)-AVFLAFGLGL-Glu(EDANS)
IQ-PGAM5$^{20\text{-}29}$	(DABCYL)-AVFLSAVAVG-Glu(EDANS)
IQ-Smac$^{51\text{-}60}$	(DABCYL)-GVTLCAVPIA-Glu(EDANS)
PINK1$^{89\text{-}111}$	GS-^{89}AWGCAGPCGRAVFLAFGLGLGLI^{111}

Table S2. Peptide substrates used in this study

PARLΔ55 (Full length)	PINK1$^{99\text{-}108}$	PGAM5$^{20\text{-}29}$	Smac/Diablo$^{51\text{-}60}$
	n = 4	n = 5	n = 4
K_M (µM)	1.9 ± 0.4	0.47 ± 0.07	6 ±1
k_{cat} (h^{-1})	0.42 ± 0.03	0.46 ± 0.02	1.3 ± 0.1
k_{cat}/K_M ($\mu M^{-1}h^{-1}$)	0.22 ± 0.08	1.0 ± 0.3	0.3 ± 0.1

Table S3. Catalytic parameters of FL PARLΔ55-mediated cleavage of IQ-peptide substrates. Experiments were conducted in duplicate with an N=4. Values are represented as mean ±SEM.

PARLΔ77 (β-cleavage)	PINK1$^{99\text{-}108}$	PGAM5$^{20\text{-}29}$	Smac/Diablo$^{51\text{-}60}$
	n = 4	n = 5	n = 4
K_M (µM)	3.3 ± 0.5	1.6 ± 0.2	5 ± 1
k_{cat} (h^{-1})	1.3 ± 0.1	0.98 ± 0.04	1.2 ± 0.1
k_{cat}/K_M ($\mu M^{-1}h^{-1}$)	0.4 ± 0.1	0.6 ± 0.1	0.3 ± 0.1

Table S4. Catalytic parameters of β-cleavage PARLΔ77-mediated cleavage of IQ-peptide substrates. Experiments were conducted in duplicate with an N=4. Values are represented as mean ±SEM.

PARLΔ55 (Full length)	PINK1$^{99-108}$	PGAM5$^{20-29}$	Smac/Diablo$^{51-60}$
	n = 3	n = 3	n = 3
K_M (μM)	13±2	1.3±0.2	3.9±0.7
k_{cat} (h^{-1})	9±0.6	4.8±1.2	5.5±0.3
k_{cat}/K_M ($\mu M^{-1}h^{-1}$)	0.7±0.1	3.6±0.6	1.4±0.3

Table S5. Catalytic parameters of FL PARLΔ55 in PLs specific activity for IQ-peptide substrates. Experiments were conducted in duplicate with an N=4. Values are represented as mean ±SEM

PARLΔ77 (β-cleavage)	PINK1$^{99-108}$	PGAM5$^{20-29}$	Smac/Diablo$^{51-60}$
	n = 4	n = 5	n = 4
K_M (μM)	8±1	0.8±0.2	17±4
k_{cat} (h^{-1})	28±2	21±1	58±7
k_{cat}/K_M ($\mu M^{-1}h^{-1}$)	3.5±0.6	26±8	3.4±1

Table S6. Catalytic parameters of β-cleavage PARLΔ77 specific proteolytic activity in PLs for IQ-peptide substrates. Experiments were conducted in duplicate with an N=4. Values are represented as mean ±SEM.

PARLΔ77 (β-cleavage)	PINK1$^{99-108}$	PGAM5$^{20-29}$	Smac/Diablo$^{51-60}$
	n=3	n=3	n=3
K_M (μM)	14±5	8±3	3.8±1
k_{cat} (h^{-1})	7±1	9±2	4.8±0.3
k_{cat}/K_M ($\mu M^{-1}h^{-1}$)	0.5±0.2	1.0±0.3	1.2±0.3

Table S7. Catalytic parameters of β-cleavage PARLΔ77 specific proteolytic activity in POPE/POPC proteoliposomes (lacking CL). Experiments were conducted in duplicate with an N=4. Values are represented as mean ±SEM.

A

Fold change (PARL in DDM) PARLΔ77/PARLΔ55	k_{cat}	K_m/k_{cat}
PINK1$^{99\text{-}108}$	3	2
PGAM5$^{20\text{-}29}$	2	0.6
Smac/Diablo$^{51\text{-}60}$	1	1

B

Fold change (PARL in PLs) PARLΔ77/PARL55	k_{cat}	K_m/k_{cat}
PINK1$^{99\text{-}108}$	3	5
PGAM5$^{20\text{-}29}$	4	7
Smac/Diablo$^{51\text{-}60}$	10	2

Table S8. The fold change in turnover rate and catalytic efficiency is shown between FL PARLΔ55 and β-cleavage PARLΔ77 either in **A.** detergent and **B.** reconstituted in proteoliposomes.
